# Differences in morphology and in composition and release of parotoid gland secretion in introduced cane toads (*Rhinella marina*) from established populations in Florida, USA

**DOI:** 10.1002/ece3.7118

**Published:** 2020-12-15

**Authors:** Steven T. Gardner, Megen Kepas, Casey R. Simons, Logan M. Horne, Alan H. Savitzky, Mary T. Mendonça

**Affiliations:** ^1^ Department of Biological Sciences Auburn University Auburn Alabama USA; ^2^ Department of Biology Utah State University Logan Utah USA; ^3^ Department of Chemistry and Biochemistry Utah State University Logan Utah USA; ^4^ Department of Biological Sciences University of Texas at El Paso El Paso Texas USA

**Keywords:** bufadienolide, epinephrine, invasion, phenotypic plasticity, *Rhinella marina*, sympathetic sensitivity

## Abstract

Cane toads are highly toxic bufonids invasive in several locations throughout the world. Although physiological changes and effects on native predators for Australian populations have been well documented, Florida populations have received little attention. Cane toads were collected from populations spanning the invaded range in Florida to assess relative toxicity, through measuring morphological changes to parotoid glands, likelihood of secretion, and the marinobufagenin (MBG) content of secretion. We found that residual body indices increased in individuals from higher latitude populations, and relative parotoid gland size increased with increasing toad size. There was no effect of latitude on the allometric relationship between gland size and toad size. We observed an increase in likelihood of secretion by cane toads in the field with increasing latitude. Individuals from southern and northern populations did not vary significantly in the quantity of MBG contained in their secretion. Laboratory‐acclimated cane toads receiving injections of epinephrine were more likely to secrete poison with increasing dose, although there was no difference in likelihood of secretion between southern and northern populations. This suggests that differences between populations in the quantities of epinephrine released in the field, due to altered hypothalamic sensitivity upon disturbance, may be responsible for the latitudinal effects on poison secretion. Our results suggest that altered pressures from northward establishment in Florida have affected sympathetic sensitivity and defensive mechanisms of cane toads, potentially affecting risk to native predators.

## Graphical abstract

Predators in the United States have evolved in sympatry with native bufonids. Compared to Australia, little attention has been paid to gland morphology or toxicity of invasive cane toads in Florida. Although we found no effect of locality on gland morphology or poison composition, our results indicate that northern FL populations show an increased likelihood of secreting poison when disturbed, and lab‐acclimated toads injected with epinephrine were more likely to secrete poison with increasing dose. This indicates sympathetic sensitivity has been altered in cane toads from establishment in northern FL.

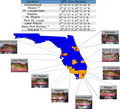

## 1 INTRODUCTION

Cane toads (*Rhinella marina*) are large and highly toxic members of the family Bufonidae and are native to South America (Acevedo et al., 2016; Zug & Zug, 1979). Due to their voracious feeding responses, cane toads were introduced into Australia in the early to mid‐1900s as a means of controlling sugar cane pests (Lever, 2001; Phillips et al., 2006) and were released in Florida (Miami) in the United States prior to 1955 (Krakauer, 1968). They have dispersed in these locations with invasion rates of up to 55 km/year in Australia (Phillips et al., 2007). Additionally, *R. marina* reproduce in large numbers, and females may lay up to 30,000 eggs per clutch in invaded areas (Hagman & Shine, 2006). However, the extent of cane toad dispersal and reproductive capacity in the United States, where they have established in higher latitude temperate areas (Mittan & Zamudio, 2019) is not well known.

Much of the devastation to native Australia fauna and predators caused by invasive cane toads has been due to their highly toxic secretions, common to bufonids and many other amphibians (Garg et al., 2007), that significantly impact the survivorship of native predators from the areas they invade (Shine, 2010). Toad poison is synthesized and stored in the large parotoid glands on the shoulders (Mailho‐Fontana et al., 2014) and in other cutaneous macroglands in the skin (Mailho‐Fontana et al., 2018). The secretion contains a cocktail of toxins (Hayes et al., 2009), the main components of which belong to a family of compounds known as bufadienolides (BDs) (Chen & Kovaříková, 1967; Lever, 2001). These steroidal compounds, derived from cholesterol (Porto & Gros, 1971), are more broadly classified as cardiotonic steroids (Sousa et al., 2017; Steyn & van Heerden, 1998). These compounds exert their effects by binding to the enzyme Na^+^/K^+^‐ATPase to induce sustained contraction. Marinobufagenin (MBG) is a potent cardiotonic compound found in the secretions of *R. marina* (Sciani et al., 2013) and has been shown to induce cell death in cardiac myocytes (Liu et al., 2012).

Altered selective pressures may affect various physiological aspects in amphibians, including the potency of poisonous secretions. Members of the toad species *Bufo bufo* occupying regions of elevated anthropogenic disturbance differ in toxicity compared to those in less disturbed agricultural areas (Bókony et al., 2019), and individuals of *Bufo boreas* exposed to increased predation cues prior to metamorphosis had higher concentrations of more toxic compounds in their secretions (Benard & Fordyce, 2003). Altered morphological and physiological changes have been documented in *R. marina* at the invasion front when compared to longer established populations (Friesen & Shine, 2019; Gardner et al., 2020). Parotoid gland size has been shown to increase with body size (snout‐vent length (SVL)) in *R. marina* (Phillips et al., 2007; Phillips & Shine, 2005). In Australia, this allometric increase is greater in toads at the invasion front than in long‐established populations (Phillips & Shine, 2005). This may indicate that cane toads produce more toxic secretions when invading new habitats, possibly to deter predators early in the invasion process, as many predators rapidly learn to avoid preying on toads (Greenlees et al., 2010).

Prior to the introduction of *R. marina*, Australia had no toad species (Beckmann & Shine, 2009; Hayes et al., 2009). In contrast, potential predators that may encounter cane toads invading Florida have evolved in sympatry with other toad species, such as the southern toad, *Anaxyrus terrestris*, which also possess BDs in their toxic secretions (Mohammadi et al., 2016). Invertebrate predators in Florida are capable of consuming cane toad eggs with low mortality risk (Punzo & Lindstrom, 2001) relative to larval anuran predators and fish. Other native predators, including several snake species, opossums, and birds (Meshaka, 2011) have been observed consuming adult toads with little or no ill‐effect.

Individuals from edge populations of *R. marina* in Australia have shown altered behavior to novel stressors, such as decreased escape behavior and an increased likelihood of secreting poison during simulated predation events (Hudson et al., 2017). Although these behaviors have yet to be observed for toads in Florida, toads near the northern edge populations have shown elevated baseline corticosterone concentrations and attenuated responses to a restraint challenge when compared to individuals from southern core populations (Assis et al., 2020). This pattern of attenuated response to novel stressors has also been documented under laboratory conditions for Florida cane toads (Gardner et al., 2020).

Although the risk of *R. marina* poisoning among native predators in Australia has been well documented (Crossland et al., 2008; Greenlees et al., 2006; Letnic et al., 2008; Phillips & Shine, 2006), that risk has not been reported quantitatively for the Florida cane toad invasion. Here, we assess morphological, behavioral, and physiological characteristics reflecting toxicity risk of *R. marina* from well‐established sites in southern Florida and from sites stretching northward to the invasion front. We measured the size of the parotoid glands, the likelihood of secretion, and the concentration of MBG in samples of secretion obtained in the field. We then collected toads to assess the epinephrine‐induced secretion response under controlled laboratory conditions. We chose to use MBG concentration as an index of overall toxicity of cane toads from field samples obtained from southern and northern populations due to the potency and abundance of this compound in the integumentary secretion of *R. marina*. We also document morphological and physiological differences between the source populations in southern Florida and established populations occurring closer to the invasion front in northern Florida.

## 2 METHODS

### 2.1 Field sampling, 2018

**Figure 1:**
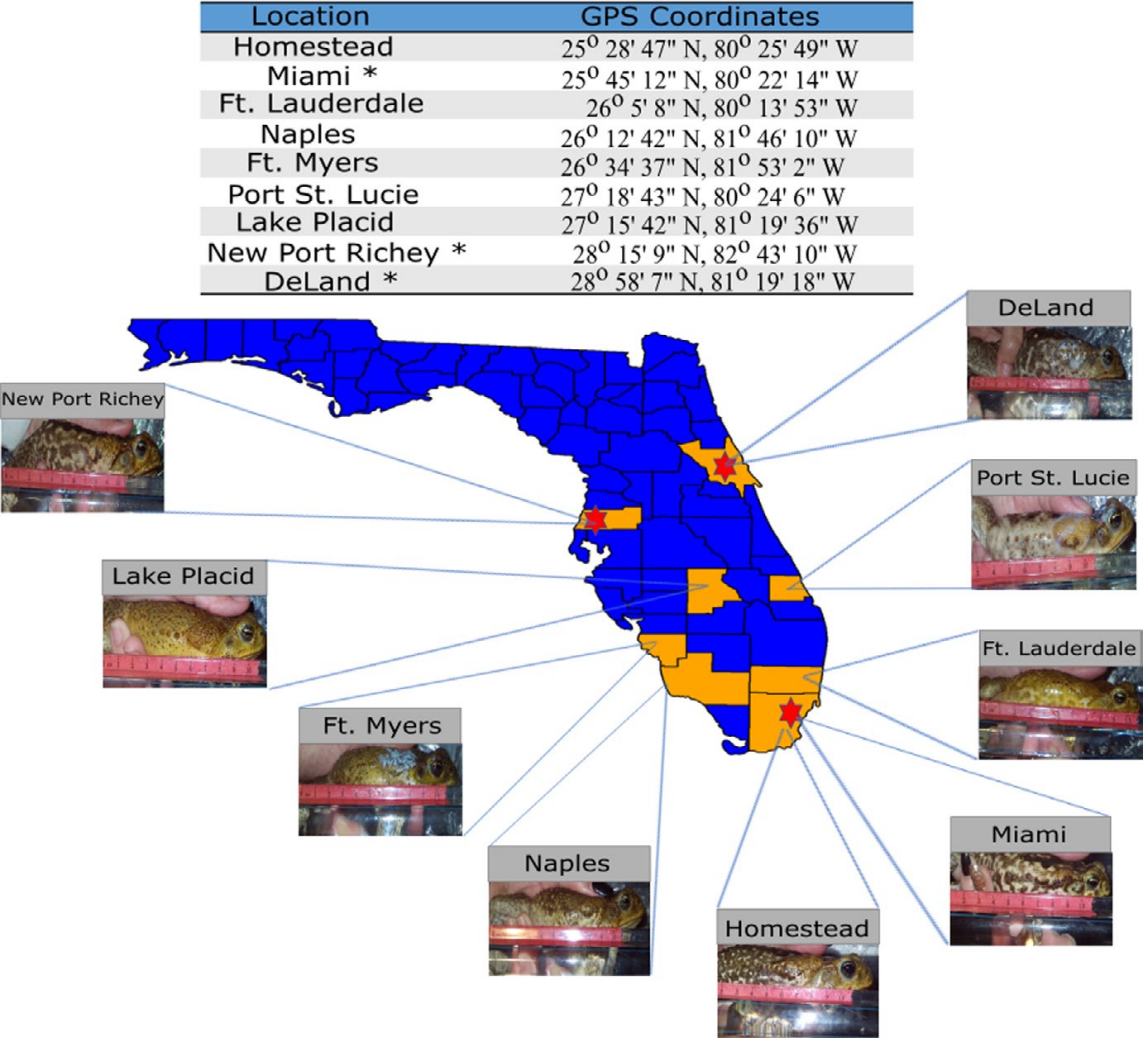
Cane toad populations sampled in 2018 for morphological measures and likelihood of secretion. Locations are listed above images of collected cane toads, with images depicting representative toads from each sampled location being recorded for gland sizes following mass, sex, and SVL being recorded. Toads were collected along a south to north gradient, from Homestead to DeLand, FL. Sites indicated by a star indicate populations sampled in 2019 for MBG concentration in poison secretions

Approximately 20 toads from nine populations (established from 1955 to 1991 (U. S. Geological Survey, 2020)) across a south–north latitudinal gradient representing the current invasive range in Florida were collected from May 10 to 19, 2018. Toads were collected from approximately 2,000–2,400 hr and placed into plastic bags immediately upon capture (Figure 1) (for exact numbers collected from each population, see Table 1). One hour following capture, toads were removed from the bags and their mass, sex, and snout‐vent‐length (SVL) were recorded. Toads were then photographed next to a ruler for scale, and the images were used to measure gland size (right parotoid gland), as well as to indicate whether a toad was secreting poison following the capture and handling period. Measurements of total length, width, and area of glands were performed using ImageJ software.

**Table 1:** Cane toad morphological data (2018)

Variable	Location	Number of individuals	Range	Median	Mean	St. dev	St. err
Mass (g)	Homestead	21	70.00 to 292.00	137.00	141.43	57.18	12.48
	Miami	20	70.00 to 271.00	118.50	131.40	50.55	11.30
	Ft. Lauderdale	20	44.00 to 158.00	93.50	98.70	31.5	7.04
	Naples	20	53.00 to 211.00	112.00	109.60	39.06	8.73
	Ft. Myers	25	76.00 to 165.00	117.00	119.72	28.64	5.73
	Port St. Lucie	20	38.00 to 235.00	102.50	110.90	44.6	9.97
	Lake Placid	20	53.00 to 150.00	90.50	96.25	30.48	6.82
	New Port Richey	20	63.00 to 308.00	100.25	122.25	60.29	13.48
	DeLand	13	44.00 to 386.00	123.00	153.00	98.32	27.27
SVL (mm)	Homestead	21	91.90 to 148.00	111.60	112.53	13.65	2.98
	Miami	20	90.00 to 189.70	108.40	113.00	21.06	4.71
	Ft. Lauderdale	20	79.20 to 120.40	98.90	99.68	9.71	2.17
	Naples	20	87.50 to 132.10	107.20	106.50	11.94	2.67
	Ft. Myers	25	92.50 to 122.70	108.50	108.21	8.75	1.75
	Port St. Lucie	20	70.30 to 121.60	103.10	101.99	11.66	2.61
	Lake Placid	20	81.50 to 115.90	98.90	97.79	10.85	2.43
	New Port Richey	20	83.50 to 145.20	97.80	102.63	16.42	3.67
	DeLand	13	75.10 to 148.40	105.90	109.34	18.26	5.06
Residual Body Index	Homestead	21	−0.12 to 0.09	0.02	0.00	0.06	0.01
	Miami	20	−0.54 to 0.10	<0.01	−0.02	0.14	0.03
	Ft. Lauderdale	20	−0.12 to 0.07	<0.01	−0.01	0.05	0.01
	Naples	20	−0.17 to 0.07	<0.01	−0.04	0.06	0.01
	Ft. Myers	25	−0.11 to 0.05	<0.01	−0.01	0.04	0.01
	Port St. Lucie	20	−0.09 to 0.29	<0.01	0.01	0.08	0.02
	Lake Placid	20	−0.06 to 0.08	0.01	0.01	0.04	0.01
	New Port Richey	20	−0.10 to 0.13	0.05	0.04	0.05	0.01
	DeLand	13	−0.08 to 0.16	0.03	0.04	0.06	0.02
Gland width (cm)	Homestead	21	1.10 to 1.94	1.5	1.54	0.24	0.05
	Miami	20	1.06 to 1.78	1.38	1.37	0.18	0.04
	Ft. Lauderdale	20	0.81 to 1.64	1.31	1.28	0.19	0.04
	Naples	20	0.97 to 2.01	1.39	1.42	0.25	0.06
	Ft. Myers	25	1.13 to 3.30	1.41	1.51	0.42	0.08
	Port St. Lucie	20	1.04 to 1.86	1.5	1.49	0.23	0.05
	Lake Placid	20	0.94 to 1.71	1.36	1.37	0.23	0.05
	New Port Richey	20	1.06 to 2.00	1.28	1.39	0.28	0.06
	DeLand	13	0.84 to 2.05	1.25	1.31	0.33	0.09
Gland length (cm)	Homestead	21	2.10 to 4.40	2.89	2.91	0.53	0.12
	Miami	20	1.92 to 3.25	2.64	2.70	0.33	0.07
	Ft. Lauderdale	20	1.78 to 2.72	2.38	2.38	0.24	0.05
	Naples	20	2.16 to 3.09	2.48	2.56	0.27	0.06
	Ft. Myers	25	2.05 to 3.51	2.46	2.57	0.37	0.07
	Port St. Lucie	20	1.77 to 3.40	2.74	2.70	0.43	0.10
	Lake Placid	20	1.34 to 3.19	2.47	2.46	0.40	0.09
	New Port Richey	20	2.08 to 3.83	2.47	2.62	0.44	0.10
	DeLand	13	1.98 to 3.45	2.81	2.76	0.41	0.11
Gland area	Homestead	21	1.61 to 5.14	2.67	2.98	0.96	0.21
	Miami	20	1.57 to 3.68	2.23	2.39	0.60	0.13
	Ft. Lauderdale	20	1.08 to 2.82	1.93	1.94	0.47	0.11
	Naples	20	1.43 to 4.69	2.29	2.40	0.71	0.16
	Ft. Myers	25	0.93 to 3.74	2.24	2.27	0.62	0.12
	Port St. Lucie	20	1.12 to 4.31	2.64	2.67	0.82	0.18
	Lake Placid	20	1.34 to 3.47	2.22	2.23	0.60	0.13
	New Port Richey	20	1.29 to 5.21	1.83	2.30	1.01	0.23
	DeLand	13	1.21 to 4.60	2.45	2.52	0.96	0.27
Gland size	Homestead	21	−0.2 to 0.41	0.06	0.09	0.16	0.03
	Miami	20	−0.23 to 0.22	−0.03	−0.01	0.12	0.03
	Ft. Lauderdale	20	−0.42 to 0.08	−0.11	−0.12	0.12	0.03
	Naples	20	−0.24 to 0.32	−0.03	−0.02	0.13	0.03
	Ft. Myers	25	−0.24 to 2.34	−0.04	0.11	0.52	0.10
	Port St. Lucie	20	−0.39 to 0.28	0.05	0.03	0.17	0.04
	Lake Placid	20	−0.30 to 0.18	−0.03	−0.06	0.14	0.03
	New Port Richey	20	−0.30 to 0.40	−0.12	−0.05	0.19	0.04
	DeLand	13	−0.20 to 0.41	0.02	−0.01	0.19	0.05

### 2.2 Field sampling, 2019

In May of 2019, toads from a southern source population in Miami (*n* = 10), as well as two northern “edge” populations, New Port Richey (NPR) and Deland (*n* = 10 NPR, *n* = 7 DeLand), were captured. Secretion samples were collected into cryovials by gentle compression of the parotoid glands (Toledo et al., 1992) and immediately frozen in liquid nitrogen. Sex, SVL, mass, and parotoid gland measurements were recorded for each toad sampled.

### 2.3 Marinobufagenin content of samples

Samples were kept frozen from the time of field collection through their transport to the laboratory. The lids of vials containing parotoid secretion were punctured with a 16G needle and the contents freeze‐dried (LABCONCO Freezone 4.5). The dry contents were removed after 24 hr, and the dry mass of each whole sample was recorded. A portion of each sample was transferred to a glass vial and diluted with 0.5ml/mg of a 90:10 methanol/water UV grade solution. The samples were then sonicated for 30 min, rotated, and sonicated for another 15 min. Following sonication, the samples were centrifuged for 15 min at 3,000 rpm to separate undissolved solid material. The supernatant was then collected and stored for analysis in a glass instrument vial (1.5 ml) at −20°C.

High‐performance liquid chromatography (HPLC) was carried out on a Shimadzu LC‐2030, equipped with an Econsil C18 column (250 x 4.6 mm, i.d.; particle size: 10 µm) from Alltech. The column was maintained at room temperature and run at a pump speed of 1 ml/min. The mobile phases consisted of A = milli‐Q water with 0.1% trifluoracetic acid and B = acetonitrile +0.1% TFA. Gradient methodology was employed using the following method: 20% B for 1 min then raised to 42% over 42 min. It was then raised to 95% B over 0.1 and held there for 4.9 min. B was lowered to 20% in 0.1 min and held for 3.9 min to return the column to equilibrium.

A commercial standard of marinobufagenin (MBG; Cayman Chemicals, catalog # 20798) was reconstituted in methanol. The peak was first identified at 40.45 min. For verification, test samples separate from those analyzed were spiked with commercially acquired marinobufagenin, and the additional peak height was used to confirm the identity of the marinobufagenin peak using the elution protocol described.

The MBG peak for each sample occurred at approximately 40.45 min per the elution gradient described above. The area of each peak was divided by the mass of the dry sample and the peak/mass ratio was used in further analysis. Peak tailing was minimal after the optimal gradient elution protocol was established. However, area under the curve was chosen over peak height to accommodate any minor peak tailing or broadening. MBG sample concentrations were determined by the use of a four‐parameter standard curve. Using the integrated peak value and a five‐point calibration plot, the concentration of MBG (µg/mg) in solution was determined. This concentration was then back‐calculated relative to the amount of solution used for sample preparation and adjusted for the dry weight of each sample prior to extraction.

### 2.4 Sympathetic sensitivity

In May 2019, 58 additional toads (*n* = 28 Miami, and *n* = 30 NPR) were collected and maintained under laboratory conditions, detailed by Gardner et al. (2020). Following acclimation, the toads were divided into five treatment groups: normal saline solution (NSS) and NSS with 0.125 µm, 0.25 µm, 0.5 µm, or 1 µm epinephrine gram of body per mass. Each treatment group was comprised of six toads collected from Miami and NPR (with the exception of the 0.125 µm treatment group, which had only four toads from Miami). Following injections, each toad was placed in a plastic bin (60.96 x 46.99 x 40 cm) and monitored for signs of poison secretion.

### 2.5 Statistical analyses

To compare the morphological parameters in Florida populations of cane toads with those observed in a previous study by Phillips and Shine (2005), a model using the lm function in R (version 3.6.3), using latitude coordinates (decimal degrees) as a covariate and sex as a factor, was used to assess differences in log_10_‐transformed SVL measurements of toads. Log_10_‐transformed body masses of toads collected from the nine populations sampled during 2018 were then regressed against SVL measurements (also log_10_‐transformed) to obtain residual body condition index measures (Denoël et al., 2002). This measure follows a normal distribution that is independent of SVL (Plăiaşu et al., 2010) and has commonly been used to assess fitness and performance of individuals from different populations (Scheele et al., 2014; Unglaub et al., 2018). A model using sex as a factor and latitude and log_10_‐transformed SVL (toad size) as covariates was used to assess differences in body index (BI) for the 2018 dataset.

Parotoid gland sizes were evaluated by performing a principal component analysis (PCA) (using the princomp function in R) on log_10_‐transformed width, length, and area of glands obtained from images taken in the field. The first principal component, termed “gland size” and characterized by parotoid gland length, area, and width, accounted for 60.19% of the variation in gland measurements, and was used in a model (using the lm function in R) with latitude, BI, and toad size as covariates and sex as a factor, to assess differences in gland sizes among the nine populations sampled in 2018. An interaction term between toad size and latitude was originally included in this model; however, as this term was not significant (*p* > .05), it was removed. An additional model (using the glm function from the nlme package in R) using sex as a factor and BI, gland size, toad size, and latitude as covariates was used to assess likelihood of the toads secreting poison in the field during 2018. BI was obtained for toads collected in 2019 from Miami, New Port Richey, and DeLand (as well as gland measurements), and concentration of MBG in collected secretions was assessed using BI, gland sizes, and toad size as covariates and location as a factor. The likelihood of the toads secreting poison following 3 weeks of laboratory acclimation was assessed using epinephrine dose as a covariate and location as a factor for toads collected in 2019. Resulting *p* values from all analyses were adjusted using Bonferroni correction in R.

## 3 RESULTS

### 3.1 Morphology and likelihood of secretion (2018)

**Figure 2:**
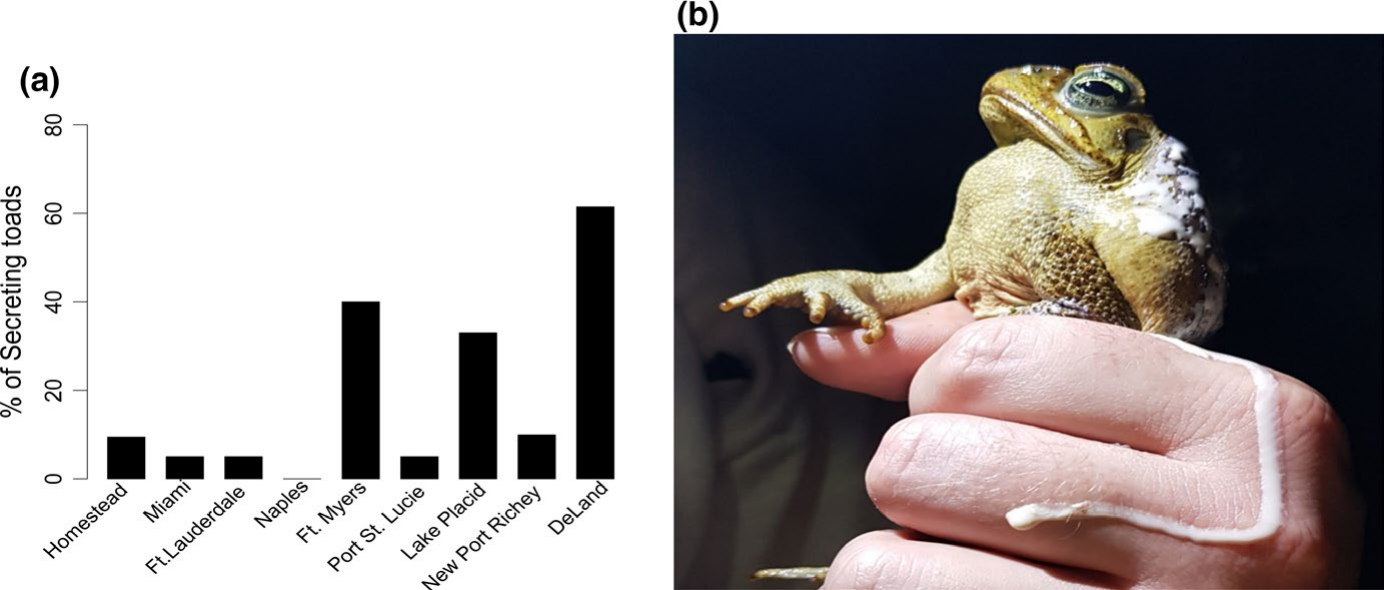
Poison secretion data collected from 9 cane toad populations spanning a south to north gradient in FL (*n* = 21, 25, and 13 for Homestead, Ft. Myers, and DeLand, and *n* = 20 for all other populations). Panel (a) represents percentages of toads secreting poison following 1 hr of capture and handling. The likelihood of a toad secreting poison following collection in the field increased significantly (z173 = 3.24, *p* < .01) with increasing latitude, from the most southern population (Homestead) to the most northern (DeLand, FL). Panel (b) depicts a toad secreting poison immediately following capture from a northern FL population (DeLand)

There was no effect of latitude (−0.008 (± 0.004), *t*_176_ = −2.06, *p* = .08) or sex (0.002 (± 0.008), *t*_176_ = 0.24, *p* = 1.0 for 110 males compared to 69 females) on sizes of cane toads collected during 2018 (values represent mean [±] standard error). BI of male toads measured in 2018 did not differ from females (−0.006 [± 0.010], *t*_175_ = −0.638, *p* = 1.0), and BI was also not affected by toad size (0.055 [±0.09], *t*_175_ = 0.59, *p* = 1.0). BI did however increase in collected toads by 0.020 (± 0.005) (*t*_175_ = 3.87, *p* < .01) with each degree of increasing latitude among the cane toad populations. Parotoid gland size was not affected by BI (0.57 [± 0.24], *t*_174_ = 2.37, *p* = .08), or latitude (−0.010 [± 0.017], *t*_174_ = 0.58, *p* = 1.0). BI was also not different for male compared to female toads (0.011 [± 0.034], *t*_174_ = 0.32, *p* = 1.0), although gland size increased significantly (1.92 [± 0.30], *t*_174_ = 6.50, *p* < .01) with increasing toad size (for the full list of sample sizes, means, standard errors, data ranges, and medians for morphological data collected in 2018 see Table 1). There was no effect of BI (0.200 (95% C.L. = 0.0005–72.84), *z*_173_ = 0.55, *p* = 1.0), gland size (2.48 [95% C.L. = 0.54–11.28], *z*_173_ = 1.20, *p* = 1.0), or toad size (2.09 [95% C.L. = 0.0007–6,022.76], *z*_173_ = 0.19, *p* = 1.0) on the likelihood of a toad to secrete poison upon capture and handling. Male toads were also no more likely to secrete poison compared to female cane toads (3.03 [95% C.L. = 1.11–8.24], *z*_173_ = 2.22, *p* = .13); however, cane toads were 1.94 (95% C.L. = 1.29–2.91; *z*_173_ = 3.24, *p* < .01, times more likely to secrete poison with each degree of increasing latitude (Figure 2).

### 3.2 MBG concentration and sympathetic sensitivity (2019)

**Figure 3:**
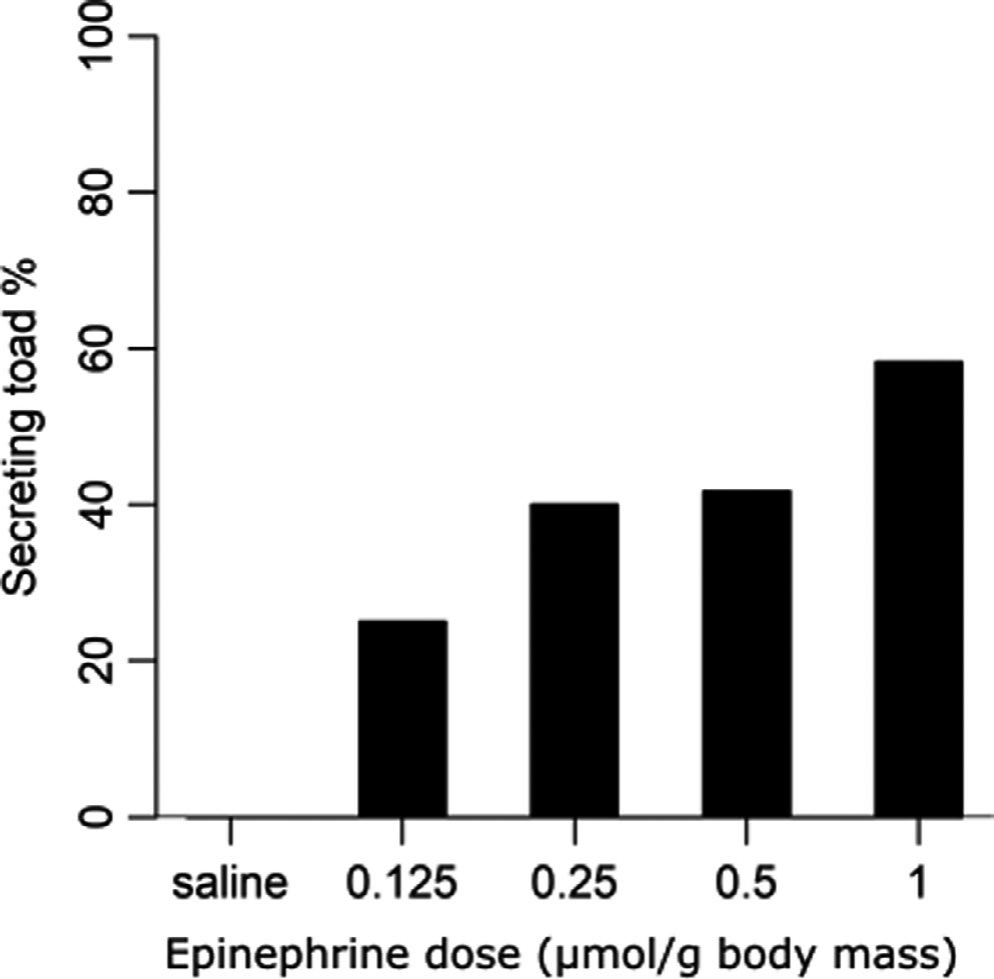
Percentages of laboratory‐acclimated cane toads collected from Miami (south) (*n* = 28) and New Port Richey (north) (*n* = 30) during 2019 secreting poison following injections with increasing doses of epinephrine (µmol ((g body mass‐1)). The likelihood of a toad secreting poison increased significantly with increasing dose (z55 = 2.71, *p* = .01), although there was no effect of location on this likelihood (*p* > .05)

MBG concentrations assessed using HPLC were not affected by BI (247.34 [± 185.45], *t*_21_ = 1.33, *p* = 1.0), toad size (469.32 [± 317.21], *t*_21_ = 1.48, *p* = .92), or gland size (−157.10 [± 100.70], *t*_21_ = −1.56, *p* = .80). MBG concentrations for Miami cane toads were not different compared to those from NPR (47.38 [± 20.87], *t*_21_ = −2.27, *p* = .21), or DeLand (−7.43 [± 29.57], *t*_21_ = 0.25, *p* = 1.0). MBG concentrations were also not different for NPR compared to DeLand cane toads (54.81 [± 30.91], *t*_21_ = 1.77, *p* = .54) (for the full list of sample sizes, means, standard errors, data ranges, and medians for morphological and MBG concentration data collected in 2019 see Table 2). Although laboratory‐acclimated toads (*n* = 28 Miami, 30 NPR) were 9.54 (95% C.L. = 1.80–50.58; *z*_55_ = 2.71, *p* = .01) times as likely to secrete poison with increasing epinephrine dose (Figure 3), the likelihood of cane toads secreting poison was not affected by locality (0.76 [95% C.L. = 0.23–2.55], *z*_55_ = −0.45, *p* = 1.0).

**Table 2:** Cane toad morphological and MBG concentration data (2019)

Variable	Location	Number of individuals	Range	Median	Mean	St. dev	St.err
Mass (g)	Miami	10	97.00 to 183.00	144.00	144.20	30.96	9.79
	New Port Richey	10	87.00 to 224.00	168.50	168.30	42.14	13.33
	DeLand	7	17.00 to 169.00	86.00	76.43	59.35	22.43
SVL (mm)	Miami	10	96.70 to 126.70	113.15	111.48	9.64	3.05
	New Port Richey	10	96.60 to 137.20	115.15	116.56	12.57	3.97
	DeLand	7	57.20 to 117.20	97.70	87.01	24.93	9.42
Residual Body Index	Miami	10	−0.12 to 0.06	<0.01	<0.01	0.05	0.02
	New Port Richey	10	−0.06 to 0.11	0.02	0.01	0.05	0.02
	DeLand	7	−0.06 to 0.01	−0.03	−0.02	0.02	0.01
Gland Width (cm)	Miami	10	1.11 to 2.06	1.63	1.57	0.28	0.09
	New Port Richey	10	1.35 to 2.04	1.79	1.78	0.20	0.06
	DeLand	7	0.88 to 1.77	1.35	1.31	0.43	0.16
Gland length (cm)	Miami	10	2.22 to 3.71	2.89	2.96	0.49	0.15
	New Port Richey	10	2.33 to 3.81	3.27	3.24	0.42	0.13
	DeLand	7	1.28 to 3.31	2.61	2.37	0.85	0.32
Gland area	Miami	10	1.78 to 4.39	2.70	3.00	0.84	0.27
	New Port Richey	10	1.80 to 4.44	3.50	3.28	0.84	0.27
	DeLand	7	0.61 to 3.37	1.92	1.94	1.22	0.46
Gland Size	Miami	10	−0.29 to 0.19	−0.07	−0.03	0.16	0.05
	New Port Richey	10	−0.26 to 0.20	0.08	0.04	0.14	0.04
	DeLand	7	−0.75 to 0.15	−0.14	−0.25	0.39	0.15
MBG (µg/g dry secretion)	Miami	10	44.2 to 164.10	131.50	123.40	38.83	12.28
	New Port Richey	10	5.3 to 153.50	80.75	74.30	49.74	15.73
	DeLand	7	23.8 to 188.50	131.90	113.37	52.30	19.77

## 4 DISCUSSION

The cane toad range we examined in Florida extends approximately 480 km northward from the original introduction site in southern Florida. Previous studies have indicated that cane toad size (log_10_‐transformed SVL) and relative parotoid gland size increases in populations near the expanding edge of the invaded range in Australia (Phillips & Shine, 2005), where cane toads have established approximately 1,600 km to the northwest (Brown et al., 2015) and 2,200 km to the southeast (Urban et al., 2008) since their original introduction in 1935. We found that neither cane toad size nor the relationship with gland size was affected by latitude for Florida populations. Although initially our results seem contradictory to the results of Phillips and Shine (2005), the time since colonization for these populations ranges from approximately 65 years for the Homestead and Miami populations to 29 years for the northern populations such as NPR (U. S. Gological Survey, 2020). Although it is unclear from our current data if the Florida populations initially showed the same pattern of increased size and gland morphology upon their initial establishment throughout the state (as time since colonization was shown to significantly affect body size and gland morphology in invasive Australian populations), it is possible that the Florida populations we sampled have now been present for long enough that increased body or parotoid gland sizes no longer afford a selective advantage (Phillips & Shine, 2005).

Unlike body and parotoid gland sizes, we did observe a significant effect of latitude on BI. Studies comparing growth rates of amphibians against latitudinal or altitudinal gradients have shown that individuals from higher latitude populations show higher growth efficiency compared to those from lower latitudes (Lindgren & Laurila, 2005), perhaps as a mechanism to mitigate the effects of cooler temperatures in higher latitudes (Berven, 1982). In addition, invasive species have been postulated to escape from the pathogens and parasites of their native range upon introduction to novel habitats (Lee and Klasing 2004), with newly invading populations having a lower parasitic load due to less “carry‐over” (Torchin et al., 2003). This “lag” has been observed in Australian cane toad populations undergoing expansion (Phillips et al., 2010). Although the effects of parasitic burden on BI in amphibians are variable (Moretti et al., 2014, 2017), in Australia the cane toad parasite *Rhabdias pseudosphaerocephala* has been shown to exert weaker effects on infected individuals nearest to the expanding edge than in longer established populations (Phillips et al., 2010). Although parasitic burdens have not been well documented in the cane toad populations of Florida, altered burdens and other pressures associated with northward establishment could further explain the elevated BI in the northern populations.

Although we found no differences in parotoid gland size relative to body size in toads from different populations we sampled from Florida, there was an increase in the likelihood that cane toads from higher latitudes would secrete poison upon handling or disturbance. Physiologically elevated glucocorticoid levels induce phenylethanolamine‐*N*‐methyltransferase (PNMT) (an enzyme in chromaffin cells of the adrenal) to synthesize epinephrine from norepinephrine (Wurtman, 2002). Baseline corticosterone levels of the cane toads collected in 2018 were shown to increase with increasing latitude (Assis et al., 2020). Elevated corticosterone levels may have contributed to the amount of epinephrine available stored and subsequently released upon capture in toads from this study and therefore to the likelihood of their secreting poison in the field. Mechanisms such as modified methylation patterns (Szyf et al., 2005) or altered receptor expression (Martin et al., 2017) that modulate sympathetic sensitivity are affected by early life experiences (Yao et al., 2008). As development of cane toad tadpoles occurs more slowly in cooler temperatures (Wijethunga at al. 2016), and tadpoles have been observed to readily cannibalize each other during development (Crossland and Shine, 2011), prolonged development or other altered selection pressures on northern Florida populations may underlie these sympathetic differences. Additionally, Australian cane toads replenishing secretions following manual compression of the parotoid glands were shown to disperse more slowly than individuals that had not secreted poison (Blennerhassett et al., 2019). This combined with the increased number of potential predators (Meshaka, 2011; Punzo & Lindstrom, 2001) may contribute to range limits of cane toads in the United States.

We found no differences in MBG concentrations of cane toads from northern populations (NPR and Deland) compared to the southern Miami population. Adults have been shown to possess five primary compounds (arenobufagin, bufalin, marinobufagin, resibufagenin, and telocinobufagin) (Hayes et al., 2009). These compounds have differing toxicities, with bufalin and telocinobufagin having higher IC_50_ values relative to the other BDs (Kamano et al., 1998). Captive‐raised Australian cane toads exposed to nonlethal predator cues during larval development were shown to shift poison content toward increased amounts of bufalin compared to other compounds following metamorphosis, suggesting investment into synthesis of more lethal compounds in the presence of stronger predation cues (Hagman et al., 2009). Further research evaluating whether synthesis of more lethal compounds is higher in northern populations, complementing the higher likelihood of secretion when disturbed, would be of interest.

While the likelihood of secretion in laboratory‐acclimated toads collected during 2019 increased with increasing doses of epinephrine, there was no difference in likelihood of secretion between northern (NPR) and southern (Miami) toad populations. The similarities in sympathetic sensitivity may have resulted from the toads being removed from the field and acclimated to the same laboratory environment prior to epinephrine injection, although altered corticosterone responses to novel stressors were still observed between toads from these populations in the study of metabolism and immune response by Gardner et al. (2020). Further studies to assess whether differences in sympathetic sensitivity between northern and southern populations are related to epinephrine release could help to elucidate the responses to injected epinephrine.

The results of this study indicate that differences in sympathetic sensitivity have arisen in cane toad populations experiencing northward dispersal in Florida. It remains unclear whether BD concentrations have been affected by the northern latitude or whether there are trade‐offs between MBG and other BDs in the parotoid gland secretion. As MBG was the only BD we examined, future studies quantifying a more diverse array of BDs in cane toad secretions are needed to more accurately assess overall toxicity, and the resulting risk to native predators. Quantifying methylation patterns from populations spanning the invaded range in Florida and assessing factors leading to the differences in sympathetic sensitivity would also provide further insights, as would ecological studies assessing predation risk. Greater sampling of individuals across life‐history stages is also necessary to determine how the volume and composition of secretion changes ontogenetically or by region.

## CONFLICT OF INTEREST

None declared.

## AUTHOR CONTRIBUTION

Steven T Gardner: Conceptualization (lead); Formal analysis (lead); Investigation (equal); Methodology (equal); Visualization (lead); Writing‐original draft (lead); Writing‐review & editing (equal). Megen Kepas: Conceptualization (supporting); Formal analysis (supporting); Investigation (equal); Methodology (equal); Validation (equal); Writing‐review & editing (equal). Casey Simons: Investigation (supporting); Methodology (supporting); Validation (equal); Writing‐review & editing (supporting). L M Horne: Investigation (supporting); Methodology (supporting); Writing‐review & editing (supporting). Alan H. Savitzky: Conceptualization (supporting); Methodology (supporting); Project administration (equal); Supervision (equal); Writing‐review & editing (equal). Mary T. Mendonca: Conceptualization (supporting); Methodology (supporting); Project administration (equal); Supervision (equal); Writing‐review & editing (equal).

## Data Availability

The data and files supporting the results are archived in Mendeley Data: https://doi.org/10.17632/xr6rpmnc75.1.

## References

[ece37118-bib-0001] Acevedo, A. A. , Lampo, M. , & Cipriani, R. (2016). The cane or marine toad, *Rhinella marina* (Anura, Bufonidae): Two genetically and morphologically distinct species. Zootaxa, 4103, 574–586. 27394759 10.11646/zootaxa.4103.6.7

[ece37118-bib-0002] Assis, V. R. , Gardner, S. T. , Smith, K. M. , Gomes, F. R. , & Mendonça, M. T. (2020). Stress and immunity: Field comparisons among populations of invasive cane toads in Florida. J Exp Zool A, 10.1002/jez.2389 32488987

[ece37118-bib-0003] Beckmann, C. , & Shine, R. (2009). Impact of invasive cane toads on Australian birds. Conservation Biology, 23, 1544–1549. 19508674 10.1111/j.1523-1739.2009.01261.x

[ece37118-bib-0004] Benard, M. F. , & Fordyce, J. A. (2003). Are induced defenses costly? Consequences of predator‐induced defenses in western toads, *Bufo boreas* . Ecology, 84, 68–78.

[ece37118-bib-0005] Berven, K. A. (1982). The genetic basis of altitudinal variation in the wood frog *Rana sylvatica* II. An experimental analysis of larval development. Oecologia, 52, 360–369. 28310396 10.1007/BF00367960

[ece37118-bib-0006] Blennerhassett, R. A. , Bell‐Anderson, K. , Shine, R. , & Brown, G. P. (2019). The cost of chemical defense: The impact of toxin depletion on growth and behavior of cane toads (*Rhinella marina*). Proceedings B is the Royal Society B, 286, 20190867. 10.1098/rspb.2019.0867 PMC6532526 31088275

[ece37118-bib-0007] Bókony, V. , Üveges, B. , Verebélyi, V. , Ujhegyi, N. , & Móricz, Á. M. (2019). Toads phenotypically adjust their chemical defenses to anthropogenic habitat change. Scientific Reports, 9, 3163. 30816222 10.1038/s41598-019-39587-3 PMC6395641

[ece37118-bib-0008] Brown, G. P. , Phillips, B. L. , Dubey, S. , & Shine, R. (2015). Invader immunology: Invasion history alters immune system function in cane toads (*Rhinella marina*) in tropical Australia. Ecology Letters, 18, 57–65. 25399668 10.1111/ele.12390

[ece37118-bib-0009] Chen, K. K. , & Kovaříková, A. (1967). Pharmacology and toxicology of toad venom. Journal of Pharmaceutical Sciences, 56, 1535–1541. 4871915 10.1002/jps.2600561202

[ece37118-bib-0010] Crossland, M. R. , Brown, G. P. , Anstis, M. , Shilton, C. M. , & Shine, R. (2008). Mass mortality of native anuran tadpoles in tropical Australia due to the invasive cane toad (*Bufo marinus*). Biological Conservation, 141, 2387–2394.

[ece37118-bib-0057] Crossland, M. R. , & Shine, R. (2011). Cues for cannibalism: cane toad tadpoles use chemical signals to locate and consume conspecific eggs. Oikos, 120(3), 327–332.

[ece37118-bib-0011] de Sousa, L. Q. , Machado, K. de C. , Oliveira, S. F. de C. , Araújo, L. dos S. , Monção‐Filho, E. de S. , Melo‐Cavalcante, A. A. d. C. , Vieira‐Júnior, G. M. , & Ferreira, P. M. (2017). Bufadienolides from amphibians: A promising source of anticancer prototypes for radical innovation, apoptosis triggering and Na+/K+‐ATPase inhibition. Toxicon, 127, 63–76. 28069354 10.1016/j.toxicon.2017.01.004

[ece37118-bib-0012] Denoël, M. , Hervant, F. , Schabetsberger, R. , & Joly, P. (2002). Short‐and long‐term advantages of an alternative ontogenetic pathway. Biological Journal of the Linnaean Society, 77, 105–112.

[ece37118-bib-0013] Friesen, C. R. , & Shine, R. (2019). At the invasion front, male cane toads (*Rhinella marina*) have smaller testes. Biology Letters, 15, 20190339. 31337295 10.1098/rsbl.2019.0339 PMC6684999

[ece37118-bib-0014] Gardner, S. T. , Assis, V. R. , Smith, K. M. , Appel, A. G. , & Mendonça, M. T. (2020). Innate immunity of Florida cane toads: How dispersal has affected physiological responses to LPS. Journal of Comparative Physiology B, 190, 317–327. 10.1007/s00360-020-01272-7 32189063

[ece37118-bib-0015] Garg, A. D. , Kanitkar, D. V. , Hippargi, R. V. , & Gandhare, A. N. (2007). Antimicrobial activity of skin secretions isolated from Indian toad, *Bufo melanostictus* Schneider 1799. Nature Preceedings, 1, 1.

[ece37118-bib-0016] U.S. Geological Survey (2020). Specimen observation data for Rhinella marina (Linnaeus, 1758 ). Nonindigenous Aquatic Species Database, Gainesville, FL. Retrieved from https://nas.er.usgs.gov/viewer/omap.aspx?SpeciesID=48

[ece37118-bib-0017] Greenlees, M. J. , Brown, G. P. , Webb, J. K. , Phillips, B. L. , & Shine, R. (2006). Effects of an invasive anuran [the cane toad (*Bufo marinus*)] on the invertebrate fauna of a tropical Australian floodplain. Animal Conservation, 9, 431–438.

[ece37118-bib-0018] Greenlees, M. J. , Phillips, B. L. , & Shine, R. (2010). Adjusting to a toxic invader: Native Australian frogs learn not to prey on cane toads. Behavioral Ecology, 21, 966–971.

[ece37118-bib-0019] Hagman, M. , Hayes, R. A. , Capon, R. J. , & Shine, R. (2009). Alarm cues experienced by cane toad tadpoles affect post‐metamorphic morphology and chemical defences. Functional Ecology, 23, 126–132.

[ece37118-bib-0020] Hagman, M. , & Shine, R. (2006). Spawning site selection by feral cane toads (*Bufo marinus*) at an invasion front in tropical Australia. Austral Ecology, 31, 551–558.

[ece37118-bib-0021] Hayes, R. A. , Crossland, M. R. , Hagman, M. , Capon, R. J. , & Shine, R. (2009). Ontogenetic variation in the chemical defenses of cane toads (*Bufo marinus*): Toxin profiles and effects on predators. Journal of Chemical Ecology, 35, 391–399. 19263169 10.1007/s10886-009-9608-6

[ece37118-bib-0022] Hudson, C. M. , Brown, G. P. , & Shine, R. (2017). Evolutionary shifts in anti‐predator responses of invasive cane toads (*Rhinella marina*). Behavioral Ecology and Sociobiology, 71, 134.

[ece37118-bib-0023] Kamano, Y. , Kotake, A. , Hashima, H. , Inoue, M. , Morita, H. , Takeya, K. , Itokawa, H. , Nandachi, N. , Segawa, T. , Yukita, A. , Saitou, K. , Katsuyama, M. , & Pettit, G. R. (1998). Structure–cytotoxic activity relationship for the toad poison bufadienolides. Bioorganic & Medicinal Chemistry, 6, 1103–1115. 9730247 10.1016/s0968-0896(98)00067-4

[ece37118-bib-0024] Krakauer, T. (1968). The ecology of the neotropical toad, *Bufo marinus*, in south Florida. Herpetologica, 24, 214–221.

[ece37118-bib-0058] Lee, K. A. , & Klasing, K. C. (2004). A role for immunology in invasion biology. Trends in Ecology & Evolution, 19(10), 523–529. 16701317 10.1016/j.tree.2004.07.012

[ece37118-bib-0025] Letnic, M. , Webb, J. K. , & Shine, R. (2008). Invasive cane toads (*Bufo marinus*) cause mass mortality of freshwater crocodiles (*Crocodylus johnstoni*) in tropical Australia. Biological Conservation, 141, 1773–1782.

[ece37118-bib-0026] Lever, C. (2001). The cane toad: The history and ecology of a successful colonist. Westbury Academic and Scientific Pub.

[ece37118-bib-0027] Lindgren, B. , & Laurila, A. (2005). Proximate causes of adaptive growth rates: Growth efficiency variation among latitudinal populations of *Rana temporaria* . Journal of Evolutionary Biology, 18, 820–828. 16033553 10.1111/j.1420-9101.2004.00875.x

[ece37118-bib-0028] Liu, C. , Bai, Y. , Chen, Y. , Wang, Y. , Sottejeau, Y. , Liu, L. , Li, X. Lingrel, J. B. , Malhotra, D. , Cooper, C. J. , Shapiro, J. I. , Xie, Z. J. , & Tian, J. (2012). Reduction of Na/K‐ATPase potentiates marinobufagenin‐induced cardiac dysfunction and myocyte apoptosis. Journal of Biological Chemistry, 287, 16390–16398. 10.1074/jbc.M111.304451 PMC3351339 22451662

[ece37118-bib-0029] Mailho‐Fontana, P. L. , Antoniazzi, M. M. , Sciani, J. M. , Pimenta, D. C. , Barbaro, K. C. , & Jared, C. (2018). Morphological and biochemical characterization of the cutaneous poison glands in toads (*Rhinella marina* group) from different environments. Frontiers in Zoology, 15, 1–15. 30479646 10.1186/s12983-018-0294-5 PMC6251109

[ece37118-bib-0030] Mailho‐Fontana, P. L. , Antoniazzi, M. M. , Toledo, L. F. , Verdade, V. K. , Sciani, J. M. , Barbaro, K. C. , Pimenta, D. C. Sciani, J. M. , Bárbaro, K. C. , & Pimenta, D. C. (2014). Passive and active defense in toads: The parotoid macroglands in *Rhinella marina* and *Rhaebo guttatus* . Journal of Experimental Zoology A, 321, 65–77. 10.1002/jez.1838 24130001

[ece37118-bib-0031] Martin, L. B. , Kilvitis, H. J. , Thiam, M. , & Ardia, D. R. (2017). Corticosterone regulation in house sparrows invading Senegal. General and Comparative Endocrinology, 250, 15–20. 28559207 10.1016/j.ygcen.2017.05.018

[ece37118-bib-0032] Meshaka, W. E. Jr (2011). A runaway train in the making: The exotic amphibians, reptiles, turtles, and crocodilians of Florida. Herpetological Conservation and Biology, 6, 1.

[ece37118-bib-0033] Mittan, C. S. , & Zamudio, K. R. (2019). Rapid adaptation to cold in the invasive cane toad *Rhinella marina* . Conservation Physiology, 7, coy075. 30800317 10.1093/conphys/coy075 PMC6379050

[ece37118-bib-0034] Mohammadi, S. , Gompert, Z. , Gonzalez, J. , Takeuchi, H. , Mori, A. , & Savitzky, A. H. (2016). Toxin‐resistant isoforms of Na ^+^ /K ^+^ ‐ATPase in snakes do not closely track dietary specialization on toads. Proceedings of the Royal Society B, 283, 20162111. 27852804 10.1098/rspb.2016.2111 PMC5124105

[ece37118-bib-0035] Moretti, E. H. , Madelaire, C. B. , Silva, R. J. , Mendonça, M. T. , & Gomes, F. R. (2014). The relationships between parasite intensity, locomotor performance, and body condition in adult toads (*Rhinella icterica*) from the wild. Journal of Herpetology, 48, 277–283.

[ece37118-bib-0036] Moretti, E. H. , Titon, B. , Madelaire, C. B. , Arruda, R. , Alvarez, T. , & Gomes, F. R. (2017). Behavioral, physiological and morphological correlates of parasite intensity in the wild Cururu toad (*Rhinella icterica*). The International Journal for Parasitology, 6, 146–154. 28725553 10.1016/j.ijppaw.2017.06.003 PMC5502792

[ece37118-bib-0037] Phillips, B. L. , Brown, G. P. , Greenlees, M. , Webb, J. K. , & Shine, R. (2007). Rapid expansion of the cane toad (*Bufo marinus*) invasion front in tropical Australia. Austral Ecology, 32, 169–176.

[ece37118-bib-0038] Phillips, B. L. , Brown, G. P. , Webb, J. K. , & Shine, R. (2006). Invasion and the evolution of speed in toads. Nature, 439, 803. 16482148 10.1038/439803a

[ece37118-bib-0039] Phillips, B. L. , Kelehear, C. , Pizzatto, L. , Brown, G. P. , Barton, D. , & Shine, R. (2010). Parasites and pathogens lag behind their host during periods of host range advance. Ecology, 91, 872–881. 20426344 10.1890/09-0530.1

[ece37118-bib-0040] Phillips, B. L. , & Shine, R. (2005). The morphology, and hence impact, of an invasive species (the cane toad, *Bufo marinus*: Changes with time since colonization. Animal Conservation, 8, 407–413.

[ece37118-bib-0041] Phillips, B. L. , & Shine, R. (2006). Allometry and selection in a novel predator–prey system: Australian snakes and the invading cane toad. Oikos, 112, 122–130.

[ece37118-bib-0042] Plăiaşu, R. , Hartel, T. , Băncilă, R. I. , Cogălniceanu, D. , & Smets, J. (2010). Comparing three body condition indices in amphibians: A case study of yellow‐bellied toad *Bombina variegata* . Amphibia‐Reptilia, 31, 558–562.

[ece37118-bib-0043] Porto, A. M. , & Gros, E. G. (1971). Biosynthesis of the bufadienolide marinobufagin in toads *Bufo paracnemis* from cholesterol‐20‐14C. Experientia, 27, 506. 10.1007/BF02147562 5132572

[ece37118-bib-0044] Punzo, F. , & Lindstrom, L. (2001). The toxicity of eggs of the giant toad, *Bufo marinus* to aquatic predators in a florida retention pond. J Herpetol, 35, 693–697.

[ece37118-bib-0045] Scheele, B. C. , Boyd, C. E. , Fischer, J. , Fletcher, A. W. , Hanspach, J. , & Hartel, T. (2014). Identifying core habitat before it’s too late: The case of *Bombina variegata*, an internationally endangered amphibian. Biodiversity and Conservation, 23, 775–780.

[ece37118-bib-0046] Sciani, J. M. , Angeli, C. B. , Antoniazzi, M. M. , Jared, C. , & Pimenta, D. C. (2013). Differences and similarities among parotoid macrogland secretions in South American toads: A preliminary biochemical delineation. Scientific World Journal, 2013, 1–9. 10.1155/2013/937407 PMC3659512 23737734

[ece37118-bib-0047] Shine, R. (2010). The ecological impact of invasive cane toads (*Bufo marinus*) in Australia. The Quarterly Review of Biology, 85, 253–291. 20919631 10.1086/655116

[ece37118-bib-0048] Steyn, P. S. , & van Heerden, F. R. (1998). Bufadienolides of plant and animal origin. Natural Products Reports, 15, 397–413. 10.1039/a815397y 9736996

[ece37118-bib-0049] Szyf, M. , Weaver, I. C. , Champagne, F. A. , Diorio, J. , & Meaney, M. J. (2005). Maternal programming of steroid receptor expression and phenotype through DNA methylation in the rat. Frontiers in Neuroendocrinology, 26, 139–162. 16303171 10.1016/j.yfrne.2005.10.002

[ece37118-bib-0050] Toledo, R. C. , Jared, C. , & Brunner, A. (1992). Morphology of the large granular alveoli of the parotoid glands in toad (*Bufo ictericus*) before and after compression. Toxicon, 30, 745–753. 1509492 10.1016/0041-0101(92)90008-s

[ece37118-bib-0051] Torchin, M. E. , Lafferty, K. D. , Dobson, A. P. , McKenzie, V. J. , & Kuris, A. M. (2003). Introduced species and their missing parasites. Nature, 421, 628–630. 12571595 10.1038/nature01346

[ece37118-bib-0052] Unglaub, B. , Steinfartz, S. , Kühne, D. , Haas, A. , & Schmidt, B. R. (2018). The relationships between habitat suitability, population size and body condition in a pond‐breeding amphibian. Basic and Applied Ecology, 27, 20–29.

[ece37118-bib-0053] Urban, M. C. , Phillips, B. L. , Skelly, D. K. , & Shine, R. (2008). A toad more traveled: The heterogeneous invasion dynamics of cane toads in Australia. American Naturalist, 171, E134–E148. 10.1086/527494 18271722

[ece37118-bib-0054] Wurtman, R. J. (2002). Stress and the adrenocortical control of epinephrine synthesis. Metabolism, Clinical and Experimental, 51, 11–14. 12040535 10.1053/meta.2002.33185

[ece37118-bib-0055] Yao, M. , Hu, F. , & Denver, R. J. (2008). Distribution and corticosteroid regulation of glucocorticoid receptor in the brain of *Xenopus laevis* . The Journal of Comparative Neurology, 508, 967–982. 18399546 10.1002/cne.21716

[ece37118-bib-0056] Zug, G. R. , & Zug, P. B. (1979). The marine toad, Bufo marinus: A natural history resume of native populations. Smithsonian Contributions to Zoology 284, 58.

